# New Oral Anaticoagulant Prescribing Decisions amongst General Practitioners: Handle with Care

**DOI:** 10.36469/9798

**Published:** 2017-05-31

**Authors:** Ann Kirby, Aileen Murphy, Colin Bradley

**Affiliations:** 1 Cork University Business School University College Cork, Cork, Ireland; 2 Department of General Practice, School of Medicine University College Cork, Cork, Ireland

**Keywords:** education, prescribing behaviour, atrial fibrillation, general practitioners, oral anticoagulants

## Abstract

**Background:** Prescribing oral anticoagulants for atrial fibrillation patients is becoming more challenging as more alternatives enter the market. While warfarin has dominated the market it is a challenging medicine to use owing to its narrow therapeutic range, increased bleeding risk and requirement for continuous monitoring. The introduction of new oral anticoagulants (NOACs) offers a wider choice but they are more costly and their use also brings additional pharmacological considerations.

**Objective:** This paper investigates if the identified risk factors (renal impairment, hepatic impairment, other co-morbidities & drug interactions) influence GPs’ NOAC prescribing decisions, using a multivariate probit model, while controlling for other GP characteristics.

**Methods:** Employing primary data, collected using a dedicated survey of Irish GPs in November 2015, a multivariate probit is employed. This measures the joint decision making process of prescribing a NOAC based on four risk factors - renal impairment, hepatic impairment, other comorbidities and drug interactions.

**Results:** Younger GPs are more likely to consider ‘other co-morbidities’ and ‘renal impairment’ as important when making NOAC prescribing decisions. Male GPs are more likely to consider ‘other co-morbidities’ and ‘drug interactions’ as important when prescribing NOACs compared to female GPs. Prescribers who have initiated NOACs are more likely to consider ‘renal impairment’ as important compared with non-initiators.

**Conclusions:** Our study highlights the importance for general practitioners prescribing NOACs and caring for patients on oral anticoagulants, of adequate education, of appropriate patient selection and of appropriate monitoring of such patients. While warfarin prescribing remains predominant, NOAC prescribing is increasing. Incorporating the risk factors into prescribing decisions signals responsible prescribing for atrial fibrillation patients. Existing prescribing guidelines/toolkits need to be used in an effective manner.

## BACKGROUND

Over the last decade the demand for pharmaceutical products (and other treatments) has risen as populations continue to age, chronic diseases become more prevalent and medical innovations are ongoing. One therapeutic area that has been revolutionised in the last decade is oral anticoagulation for the treatment of atrial fibrillation (AF). Atrial fibrillation is an abnormal heart rhythm that, if left untreated, can result in a clot from the heart travelling to the brain resulting in a stroke. While this is not the only cause of stroke, patients with AF have a five-fold increase in risk of stroke.[1,2] Furthermore the presence of AF at the time of stroke is associated with higher mortality and recurrence.[1,3]

The most widely used oral anticoagulant (OAC), prescribed for decades is warfarin. However, it is not trouble-free. It has a narrow therapeutic range meaning that the difference between a dose that stops clotting and one that causes bleeding is very small. Furthermore, the response to any given dose varies over time. This means that safe use necessitates extensive monitoring of the coagulant effect which is costly for the health care provider and patient.[4] The introduction of new anticoagulants (dabigatran, rivaroxaban, apixaban, and edoxaban, collectively referred to as NOACs or DOACs), whose safety and efficacy compared with warfarin were demonstrated in large clinical trials (no comparisons between the NOACs have been done in a large RCT), offer the possibility of overcoming the restrictions associated with warfarin. Thus they have been welcomed by patients and clinicians. However, these novel drugs are expensive and their safe and effective use still involves complex pharmacological considerations.

As it stands there are two contrasting vehicles for anticoagulation. There is the "old reliable" that has been around for years and is familiar – warfarin and then there is the "new innovative" version, NOACs. It is widely acknowledged that warfarin and each of the NOACs have unique pharmacokinetic and pharmacodynamic characteristics. This makes prescribing anticoagulants and caring for patients complex.[5] In addition the need to consider each patient's characteristics (eg, adherence) is essential.[6-8] The differences between individual patients present challenges, limitations and clinical issues which are documented in the literature.[1,9-11] It is imperative that prescribers are aware of and understand these differences as the diffusion of NOACs into clinical practice increases. In an effort to address these challenges the North American Thrombosis Forum (NATF) recently published a consensus document[12] that offers a practical guide on stroke and bleeding assessment for AF patients by synthesising all available information creating a reference for the care of AF patients. This includes prescribing and monitoring guidelines as well as updated risk protection tools. Ruff *et al.*[12] recognised that, while a barrage of information is available, it may be difficult for clinicians to synthesis and process it all while prescribing, which may impact the adoption of NOACs.

Despite the widespread publication of educational tools, information guides etc. there is concern about the gap between what prescribers and those caring for NOAC patients should know in theory and what happens in practice. Despite the wealth of information available, the publication of NATF's recent document[12] signals that consensus had not be reached previously. Furthermore, while consensus may be within reach in the US where the NOAC trials were conducted and NOAC prescribing is prominent, diffusion has been slower elsewhere, for example in Ireland.

Therefore, it is imperative that those who are prescribing OACs and caring for OAC patients receive adequate education. Furthermore, it is essential that prescribers prescribe the appropriate anticoagulant (warfarin or NOACs) on a per patient basis and provide suitable monitoring and maintenance. This may not be such a significant challenge in the hospital setting where cardiologists, haematologists etc. are on site. In the community, however, where General Practitioners (GPs) are the initial contact for patients, they may not have access to the same specialist knowledge in a timely manner.

The Irish health care system comprises of both public and private elements. Approximately 80% of all medicines are funded through general taxation and the remainder through out of pocket payments. The publicly funded medicines are funded through four schemes collectively referred to as Primary Care Reimbursement Schemes (PCRS). The largest of these schemes is General Medical Services (GMS), whereby there is a fixed user charge per item dispensed (€2.50 per item capped at maximum of €25). Patients on the GMS scheme also get free general practitioner (GP) visits and there is no income incentive on the prescribers' choice of drug. In addition, a distinct scheme for NOAC reimbursement was launched in 2013.[13]

In 2014 spending on OACs (for all indications) accounted for €14.03 million in the PCRS in Ireland[14]; of which 86.9% are for AF.[15] NOACs (dabigatran and rivaroxaban only) accounted for 88% of this spend but only 20% of total OAC prescriptions. The remaining 12% spend was on warfarin representing 80% of OAC prescriptions) in the PCRS.[14] So four times more warfarin prescriptions were dispensed at 13% of the cost. Thus warfarin remains the predominant oral anticoagulant (as is recommended by the Medicines Management Programme[8,16]). Moreover the current spend on NOACs, as a proportion of total PCRS funding, is relatively low (less than 1%) but is increasing. For example, spending on NOACs increased by 55% (€4.4 million) between 2014 and 2013 and this trend is set to continue.

Prescribing NOACs is a complex decision making process requiring the consideration of numerous factors. While this is not new, the factors to consider when prescribing warfarin are well understood and INR monitoring provides a safeguard. Ironically, while the elimination of INR monitoring with NOACs is an attractive feature, without standardised monitoring/maintenance prescribers and patients are exposed to avoidable risks. Four factors have been identified in the literature and tool kits (HSE, EHRA) as necessary to consider when prescribing NOACs: renal impairment, hepatic impairment, comorbidities and drug interactions (polypharmacy). Incorporation of these risk factors into NOAC prescribing decisions signals responsible prescribing. This paper considers how the identified risk factors (renal impairment, hepatic impairment, other comorbidities and drug interactions) and other GP characteristics influence GPs' NOAC prescribing decisions using a multivariate probit.

## METHODS

To analyse if the identified risk factors influence GP's NOAC prescribing decisions, while controlling for other GP characteristics, using a multivariate probit. This measures the joint decision making process of prescribing a new oral anticoagulant but centred on a number of different risk factors - renal impairment, hepatic impairment, other co-morbidities and drug interactions. These risk factors have been identified in the literature as important when prescribing NOACs, particularly when initiating prescribing, owing to the pharmacokinetic and pharmacodynamic characteristics of anticoagulants.[7,8] As the decision to prescribe is influenced by these factors it is important to allow for the different combinations in prescribing decisions. Each of these factors are represented as a binary variable where 1 indicates that the GP rated it as important and 0 indicates that it is not important when deciding to prescribe a new oral anticoagulant. Given clinical recommendations in the literature, a positive relationship is expected between these risk factors when deciding to prescribe a new oral anticoagulant.

Individual probit models could be used to determine which GP's characteristics influence the probability of GPs considering each factor important or not when prescribing a new oral anticoagulant. However, this would not capture the unobservable individual heterogeneity and endogeneity of the discrete explanatory variables which is taken into account by using a multivariate probit model. Multivariate probit analysis explicitly assumes that the error terms may be correlated. Rather than measuring each equation independently the error terms are assumed to follow a normal distribution. Thus the multivariate nature of this analysis allows for a deeper investigation of the association between GP characteristics and prescribing behaviours, and the correlation among different risk factors that capture GPs knowledge when deciding to prescribe NOACs.[17]

The model specified is as follows:

$Y_i^* = X_i \beta + \varepsilon_i$ (1)

Where $Y_i^*$ denotes a vector of K latent variables of risk factors in K different rankings for observation *i*, $X_i$ is a set of explanatory variables related to GP characteristics such as age, gender, where they are located, initiating prescriber, anticoagulation clinic and training practice located in the practice and $\varepsilon_i$ is the error term. The four alternatives that are ranked in order of importance when prescribing a new oral anticoagulant are identified in the literature as; renal impairment, hepatic impairment, other comorbidities and drug interactions.

$$Y_{i1}^* = X_i \beta_1 + \varepsilon_{i1} \text{ for k=1 (renal impairment) (2)}$$
$$Y_{i2}^* = X_i \beta_2 + \varepsilon_{i2} \text{ for k=2 (hepatic impairment) (3)}$$
$$Y_{i3}^* = X_i \beta_3 + \varepsilon_{i3} \text{ for k=3 (other co-morbidities) (4)}$$
$$Y_{i4}^* = X_i \beta_4 + \varepsilon_{i4} \text{ for k=4 (drug interactions) (5)}$$

Furthermore $Z_i$ denotes the vector of observed binary 0/1 responses of the *ith* observation (ie, the likelihood of them displaying correct knowledge ie, ranking the risk factors as important signalling responsible prescribing of new oral anticoagulants). This model allows us to identify a set of variables which are instrumental in identifying the effect of the latent variables on prescribing decisions. Multivariate probit analysis also assumes that the error terms may be correlated and that the four error terms follow a multivariate normal distribution with a mean of zero and a variance-covariance matrix.

As there are four correlated binary response variables (ie, renal impairment, hepatic impairment, other co-morbidities and drug interactions) the multivariate probit model is applied. This multivariate model fully exploits the correlation structure. A vector of correlated binary data can be used as a proxy for knowledge and a signal of responsible prescribing which enables an analysis of the association between prescribing anticoagulants and GP characteristics. These are estimated using the mvprobit command in the STATA 14.1 (2015) software.

**Data**

Primary data was collected using a postal survey which was distributed to 1422 Irish GPs in November 2015 (A copy of the survey is available in supplementary information). Ethical approval was granted by the University's Social Research Ethics Committee and the survey was piloted. The survey had a 22% response rate with 307 responses received. Table 1 presents summary statistics for the sample. All GP practices within that sample (91% [278]) who prescribe or treat AF patients with new oral anticoagulants were included.

Average age of GPs was 53 years (SD 13) with the youngest aged 30 and the eldest aged 80 years. 66% of the sample were male and 34% were female GPs. The majority of GPs were located in practices in an urban location (82%). Furthermore approximately one-third of GPs indicated that the practice in which they were chiefly located had dedicated anticoagulation clinics or was a GP training practice (See Table 1).

**Table 1.** Summary Statistics: GP Practices in Ireland

| | Mean /Freq | SD / % |
|---|---|---|
| Age[1] | 53 | 13 |
| NOAC Prescriber / treat | 278 | 91% |
| Initiated NOAC Prescribing | 121 | 44% |
| Apixaban[2] | 73 | 26% |
| Pradaxa[3] | 53 | 19% |
| Xarelto[4] | 110 | 40% |
| Male[5] | 184 | 66% |
| Rural | 53 | 18% |
| Urban | 228 | 82% |
| Initiating Prescriber[6] | 157 | 56% |
| Anticoagulation Clinic | 94 | 34% |
| Training Practice | 85 | 31% |
| Renal Impairment - Important | 143 | 51% |
| Hepatic Impairment - Important | 201 | 72% |
| Other co-morbidities - Important | 230 | 83% |
| Drug Interactions - Important | 207 | 74% |

NOAC: new oral anticoagulant; SD: standard deviation
Notes: n=278; Missing Values: 15 non response, 2-412 missing values, 54 non response, 63 non response

Approximately 44% of GPs indicate they have initiated the prescribing of NOACs for AF patients. With regards to ranking the identified factors as important when prescribing new oral anticoagulants, over 74% considered drug interactions as important while 83% ranked co-morbidities as important. 72% ranked hepatic impairment as important while only 51% considered renal impairment as important (See Figure 1).

**Figure 1.** Respondents Ranking of Risk Factors when Prescribing NOACs

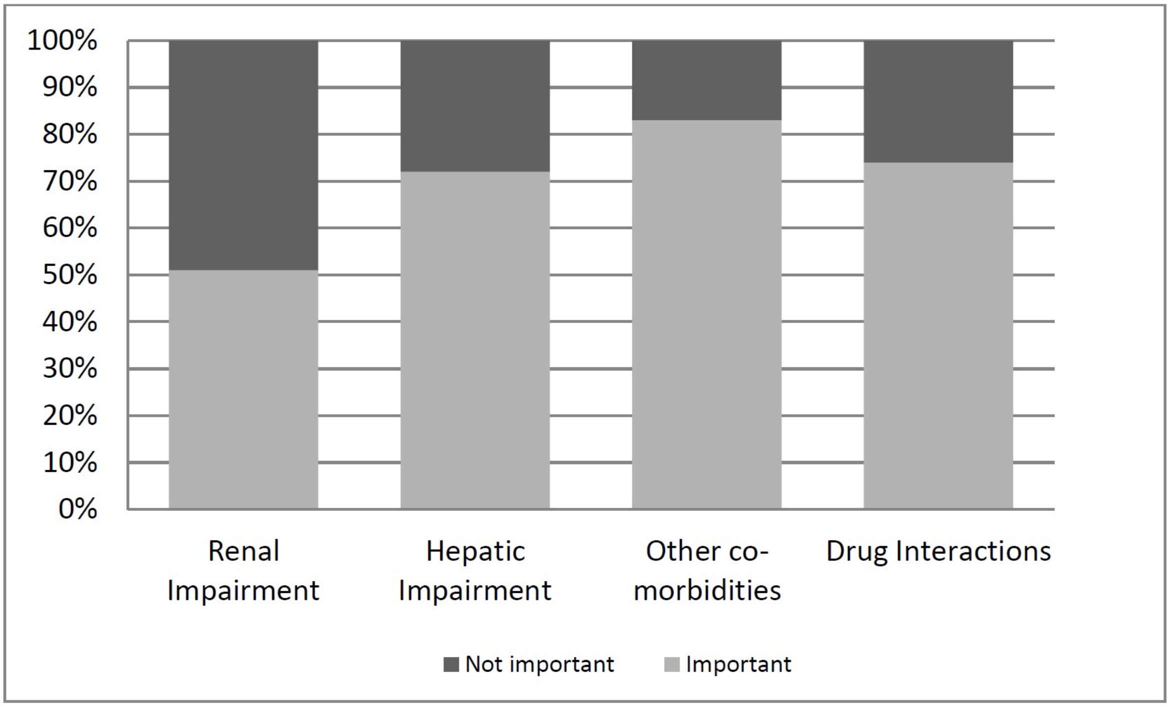

## RESULTS

The multivariate probit examines the decision making process for prescribing a new oral anticoagulant based on four risk factors (renal impairment, hepatic impairment, other comorbidities and drug interactions) which serve for the purposes of this research as a signal of GPs' prescribing responsibility. The model also controls for GP characteristics.

With regards to GP characteristics, the age of the GP is statistically significant for two patient risk factors, 'other comorbidities' and 'renal impairment'. More experienced GPs are less likely to rank 'other comorbidities' (p=0.03) and renal impairment (p=0.10) as important when prescribing new oral anticoagulants. Also, male GPs are more likely to rank 'other comorbidities' (p=0.01) and 'drug interactions' (p=0.01) as important when prescribing a new oral anticoagulant compared to female GPs. Furthermore, initiating prescribers of NOACs are more likely to rank 'renal impairment' (p=0.00), 'as important compared to prescribers who did not initiate NOACs. The three practice characteristics included in the model (location, anticoagulation clinic and training practice) were not found to be statistically significant (See Table 2).

**Table 2.** Multivariate Probit Results

| Characteristics | Renal Impairment | Hepatic Impairment | Other Comorbidities | Drug interactions |
|---|---|---|---|---|
| Age | -0.01* | -0.00 | -0.02** | -0.01 |
| | (0.01) | (0.01) | (0.01) | (0.01) |
| Male | 0.06 | 0.16 | 0.52*** | 0.49*** |
| | (0.16) | (0.16) | (0.20) | (0.17) |
| Initiating Prescriber | 0.49*** | 0.04 | 0.20 | 0.23 |
| | (0.16) | (0.16) | (0.18) | (0.17) |
| Location | 0.12 | -0.07 | 0.12 | 0.04 |
| | (0.20) | (0.21) | (0.23) | (0.21) |
| Anticoagulation clinic | 0.04 | 0.06 | 0.21 | 0.13 |
| | (0.17) | (0.18) | (0.20) | (0.19) |
| Training Practice | 0.03 | 0.06 | 0.22 | 0.16 |
| | (0.17) | (0.18) | (0.21) | (0.19) |
| Constant | 0.11 | 0.51 | 0.76* | 0.19 |
| | (0.47) | (0.48) | (0.53) | (0.49) |

Note: Notation in ( ) represent standard errors. Location refers to Urban areas
*
**
***

As anticipated, given prescribing literature, the correlation between the risk factors (renal impairment, hepatic impairment, other co-morbidities and drug interactions) is statistically significant and positive. The strongest correlation estimated is between renal and hepatic impairment (ϱ=0.75). Correlations between 'drug-interactions' and other 'co-morbidities with the other risk factors are weaker. For example, the weakest correlation is between 'drug-interactions' and 'hepatic impairment' (ϱ=0.43) (See Table 3).

**Table 3.** Variance/Co-variance Matrix of the Risk Factors

| Prescribing Risk Factors | ϱ | SE |
|---|---|---|
| Renal & Hepatic | 0 .75*** | (0.06) |
| Renal & Other comorb. | 0.47*** | (0.10) |
| Renal & Drug interactions | 0.45*** | (0.09) |
| Other Comorb. & Hepatic | 0.64*** | (0.08) |
| Drug Interactions & Hepatic | 0.43*** | (0.09) |
| Drug Interactions & Other comorb. | 0.46*** | (0.10) |

Note: Renal, Hepatic & Other comorb. are Renal Impairment, Hepatic Impairment & Other comorbidities consecutively. ϱ= rho-a measure of correlation and SE are the associated standard errors. chi2(6) = 144.591; Prob > chi2 = 0.0000
***

## DISCUSSION

New drugs increase prescribing costs and increase the risk of adverse reactions/events.[18] Many studies have analysed GP prescribing behaviour across all medicines and new drugs across a variety of public health care systems (Greece[19], United Kingdom[20-22], Ireland[23], and Sweden[24]). Specifically, previous research indicates that uptake of new drugs is influenced by doctor characteristics[25], patients[26], personal contact with colleagues, hospital consultants and pharmaceutical industry2[7,28]; acquired knowledge and differences in subjective and ideological beliefs.[18] Furthermore, prescribing behaviour by therapeutic area/ drug type is also emerging.[29-32] However, few have considered the NOAC prescribing landscape with the joint consideration of different risk factors and the prescribing decision while controlling for other GP characteristics as per this paper.

Using primary data collected amongst Irish GPs a multivariate probit was employed to determine the joint decision of what risk factors influence NOAC prescribing decisions while considering GP characteristics. With regards to GP characteristics, younger GPs are more likely to consider 'other co-morbidities' and 'renal impairment' as important when making NOAC prescribing decisions. Also, male GPs are more likely to consider 'other co-morbidities' and 'drug interactions' as important when prescribing NOACs compared to female GPs. Finally, prescribers initiating NOACs are more likely to consider 'renal impairment', as important compared to those who do not initiate prescribing of NOACs. The risk factors 'hepatic impairment', 'other co-morbidities' and 'drug interactions' were not found to be a significant risk factor when considering to prescribe a NOAC.

Given that the characteristics of the practice where the GP is located did not appear to influence prescribing, only GP characteristics were examined. In suggesting that younger GPs are more cognisant of the risks and associated responsibilities when prescribing NOACs this study complements previous research. For example, Bourke[23] showed that younger GPs are more likely to be early adopters. Furthermore, previous research[33] demonstrates that females were less likely than males to prescribe new drugs. This paper suggests findings in line with this. However we acknowledge that there were more male than female GPs in the sample (which is representative of the Irish GP population). The only other characteristic that was significant was if the GP initiated the prescribing of NOACs. Prescribers who initiated NOACs were more likely to rank one of the four patient risk factors as important when prescribing a NOAC. Furthermore the results of the survey reveal a knowledge gap between GPs who have initiated NOAC prescribing and those who have not. While GPs who have not initiated NOACs may be caring for AF patients prescribed NOACs by another doctor, previous research indicates that GPs may be reluctant to alter or discontinue a hospital-initiated prescription.[34] This difference between prescribers who have or who have not initiated NOACs signals a potential moral hazard problem amongst GPs who have not initiated NOACs but who are caring for AF patients prescribed

NOACs. GPs who have initiated NOACs may have a greater sense of responsibility towards longer term issues such as renal impairment.

As anticipated, given prescribing literature, the correlation between the risk factors (renal impairment, hepatic impairment, other co-morbidities and drug interactions) is statistically significant and positive. However correlations between 'drug-interactions' and other 'co-morbidities' with the other risk factors are weaker. A priori, a strong if not perfect correlation between routine risk factors like comorbidities was anticipated but this was not the case. This may indicate that when prescribing in a 'real world' setting the available education and decision making tools are not pragmatic enough. The identified risk factors should not be considered mutually exclusive rather they should be complementary to each other and patient centred.

As with any study reliant on primary data collected via postal survey there are limitations to this study. In the absence of a national database and particularly email addresses for respondents, a postal survey was necessary. While a 22% response rate is respectable, we acknowledge the sample size is small and could be subject to response bias. However the sample characteristics are comparable to other published GP surveys[35-36] and the OECD.[37] We have acknowledged sample size and consider the results of the study in the context of those who did respond. Also the HSE information prescribing tool was published in September 2015 which was shortly before the postal survey was issued (November 2015). Nevertheless GPs have been prescribing warfarin for decades and NOACs since 2012. We also acknowledge there are other risk factors than the four used here. The four considered here were selected based on the "Anticoagulation Prescribing Tips", EHRA anticoagulation card and response rates to the question in the survey.[7-8]

## CONCLUSIONS

As the PCRS evidence[12] suggests, warfarin remains the predominant oral anticoagulant (as is recommended by the Medicines Management Programme). Nevertheless prescribing of NOACs is continuing and is expected to expand. Thus, the two types of OACs – the old and the new will continue to co-exist. Therefore, GPs acting as agents on behalf of their patients (the principals) need to ensure they are prescribing effectively and responsibly. This study considered four risk factors which act as a proxy for knowledge and a signal of responsible prescribing. The findings suggest that while education and decision making tools are emerging they are not being used to their full potential. Prescribing NOACs for AF patients is a complex process with pharmacological issues, patient preferences and the need to consider the common good of the patient at all times. The multivariate probit results revealed that the joint decision making of prescribing NOACs while considering risk factors is currently sub-optimal. To remedy this for current and future prescribers' education and decision making tools need to be accessible and pragmatic for use in a real world setting amongst a heterogeneous population.
